# SIRT1 expression is associated with a poor prognosis, whereas DBC1 is associated with favorable outcomes in gastric cancer

**DOI:** 10.1002/cam4.310

**Published:** 2014-08-21

**Authors:** Akira Noguchi, Keiji Kikuchi, Huachuan Zheng, Hiroyuki Takahashi, Yohei Miyagi, Ichiro Aoki, Yasuo Takano

**Affiliations:** 1 Research Institute, Kanagawa Cancer CenterYokohama, Kanagawa, Japan; 2 Department of Biochemistry and Molecular Biology, College of Basic Medicine, China Medical UniversityShenyang, Liaoning, China; 3 Department of Pathology, School of Medicine, Kitasato UniversitySagamihara, Kanagawa, Japan; 4 Department of Molecular Pathology, School of Medicine, Yokohama City UniversityYokohama, Japan

**Keywords:** Cancer progression, gastric cancer, p53 independency, prognostic indicator, SIRT1 expression

## Abstract

Clinical trials of histone deacetylase (HDAC) inhibitors as antitumor therapy have been conducted for gastric cancer. Expression of SIRT1, a class III HDAC, is related to poor prognosis in some malignancies. We investigated the correlation between SIRT1 expression and progression and prognosis of gastric cancers comparing with molecules linked to SIRT1 in order to better predict the efficacy of HDAC inhibitors in treating this disease. We evaluated SIRT1 expression by western blot in 51 cases and SIRT1, DBC1, acetylated H4K16 (H4K16Ac), acetylated H3K9 (H3K9Ac), and p53 by immunohistochemistry (IHC) in 557 cases of gastric cancer. Western blotting showed that SIRT1 high expression related with statistics to advanced tumor progression, positive lymphatic invasion, positive venous invasion, and advanced stage but not to poor prognosis. IHC revealed that SIRT1 high expression correlated with worse clinico-pathological prognostic factors as same as in western blotting and related poor prognosis both by univariate and multivariate analyses. By the contrast, DBC1 and H4K16Ac were related to favorable prognostic factors and linked to favorable prognosis by univariate analysis but not by multivariate analysis. H3K16Ac correlated only favorable prognostic factors. Results of p53 were very similar to those of SIRT1. We found that SIRT1 high expression closely correlates with progression and prognosis in gastric cancer patients. And it was also indicated that SIRT1 acts as an oncogene by the results of DBC1, H4K16Ac, and H3K9Ac and might be a target molecule of HDAC inhibitor treatment for gastric cancer patients.

## Introduction

Although the incidence of gastric cancer is decreasing worldwide, its prognosis remains generally poor. Various genetic alterations, such as *K-ras* and *APC* mutations or loss of *DCC*, are thought to be involved in gastric carcinogenesis. However, recent studies have indicated that, in addition to genetic lesions, epigenetic changes such as DNA methylation and histone modification also play a crucial role in tumor initiation and malignant progression [1].

The sirtuin family is composed of seven genes (*SIRT1*–*7*) that encode NAD^+^-dependent histone deacetylases (Class III, HDACs) and are conserved from archaebacteria to eukaryotes [2]. *SIRT1* is the mammalian ortholog of the *Sir2* gene, which is linked to aging in nematodes and plays a crucial role in cell metabolism, longevity, and stress response [3]. SIRT1 represses the function of p53, KU70, and the FOXO family proteins through deacetylation, and SIRT1 downregulation induces cell cycle arrest and apoptosis in cancer cells [4]. SIRT1 upregulation has been reported in various malignant tumors in humans and animals [5]. In cancer cells, SIRT1 has been detected in the promoter of densely hypermethylated tumor suppressor genes (TSGs; e.g., *E-cadherin*, *MLH1*, and *p27*) and may contribute to their transcriptional inactivity [6]. However, it might also independently function as a TSG, since *Sirt1*^*−/−*^ mice exhibit impaired DNA damage response [7]. In addition, SIRT1 overexpression in *Apc*^Min/+^ mice induces *β*-catenin deacetylation and reduces colon tumor formation [8]. Nonetheless, it is worthy of note that such conflicting functions of SIRT1 were determined by studies of SIRT1 expression in cancer and its effects on well-known oncogenes and tumor suppressors [9].

*DBC1* was originally cloned from chromosome 8p21 that was homozygously deleted in 3.5% of breast cancers [10]. DBC1 was shown to involve in the induction of apoptosis in response to tumor necrosis factor-*α* [11]. Additionally, it has been reported as a negative regulator of SIRT1 [12, 13]. DBC1 interacts with the catalytic domain of SIRT1, inhibits its deacetylase activity, and increases p53 acetylation level, thereby enhancing p53-mediated apoptosis. However, despite the reported tumor suppressor function of DBC1, some studies have described increased expression of DBC1 in breast carcinoma [14], suggesting that DBC1 might participate in the development and progression of breast cancer. Similar phenomenon has also been observed in gastric cancer. The expression of both DBC1 and SIRT1 is reported to be associated with a poor prognosis in gastric cancer [15].

Recruitment of SIRT1 to its target gene promoter results in deacetylation of histone proteins at H1 lysine 26 (H1K26Ac), H4 lysine 16 (H4K16Ac), and H3 lysine 9 (H3K9Ac). SIRT1 not only recruits and deacetylates the suppressor of variegation 3–9 homolog 1 (SUV39H1), resulting in H3K9 trimethylation [16] but also forms a protein complex with SUV39H1 and nucleomethylin. Such a protein complex binds H3K9 in the rDNA locus and dimethylates and deacetylates H3K9, thus repressing rRNA transcription and resulting in inhibition of apoptosis [17]. Acetylation of the lysine residues within the *N*-terminal tails of histone H3 and H4 proteins generally correlates with the establishment of an open chromatin conformation that is transcriptionally active [18]. Acetylation of the histone H3 tail seems to properly control gene expression, whereas that of the H4 tail might play a role in DNA replication [19]. Since SIRT1 is connected to senescence, apoptosis inhibition, blockage of cell differentiation, and cell growth promotion, the oncogenic role of SIRT1 might be significant [9]. However, it also serves as a TSG because of its role in maintaining genome stability through chromatin regulation and DNA repair [20, 21].

Thus, DBC1 and SIRT1 might be cooperatively involved in gastric tumorigenesis via histone acetylation. A better understanding of their interaction at the protein level could potentially lead to novel therapeutic approaches in gastric cancer management. Therefore, this study aimed to investigate SIRT1 protein expression in gastric tumor specimens and correlate histone acetylation with clinicopathological parameters and patient outcome.

## Materials and Methods

### Patients and samples

For western blot analysis, cancerous and corresponding noncancerous tissues were obtained from 51 gastric cancer patients treated at the Kanagawa Cancer Center Hospital (KCCH) between August 2006 and January 2010 and stored at −90°C. For immunohistochemistry (IHC) studies, formalin-fixed and paraffin-embedded tissues were obtained from 557 cohort gastric cancer cases encountered at KCCH between January 1999 and July 2002 according to patient records. All cases underwent radical gastrectomy and pathological samples were reviewed with histological typing independently reconfirmed by 3 pathologists (Y. T., Y. K., and A. N.) according to the World Health Organization classification guidelines [22]. Adenocarcinomas were divided into well, moderately, and poorly differentiated tumor subtypes. Other clinicopathological variables, including lymphatic or vessel invasion and depth of invasion, were also reconfirmed. Depth of invasion was divided into 4 groups from T1 to T4. Pathologic staging was reviewed based on the tumor-node-metastasis staging system of the Union for International Cancer Control [23]. The mean follow-up period was 76 months (median, 69 months; range, 6–142 months). The median age of all patients was 63 years (range, 24–87 years) and the male-to-female ratio was 2.4. The local ethics committee of the Kanagawa Cancer Center approved this study. Informed consent was obtained in accordance with the Declaration of Helsinki.

### Western blot analysis of cancerous and noncancerous tissues

Western blot analyses of both cancerous and noncancerous tissues were performed. Frozen tissues were weighed and homogenized in 10–20 times volume of the Tissue Protein Extraction reagent (Thermo Scientific; Rockford, IL) supplemented with a protease inhibitor cocktail (Roche, Mannheim, Germany) using a Polytron-type homogenizer. The lysates were centrifuged at 15,000*g* for 10 min, and the supernatant was recovered. Protein concentrations were determined using Bradford reagent (BioRad Laboratories, Hercules, CA). Proteins (20 *μ*g) were resolved by electrophoresis on a 10% sodium dodecyl sulfate polyacrylamide gel and transferred onto a polyvinylidene difluoride membrane (Millipore, Bedford, MA). The membranes were incubated with primary antibodies (anti-SIRT1 rabbit mAb, clone E104, Epitomics, Burlingame, CA, 1:1000 dilution and anti-*β*-actin mouse mAb, AC74, Sigma Aldrich, St. Louis, MO, 1:1000) overnight at 4°C. After subsequent washing, they were detected using an enhanced chemiluminescence system (GE Healthcare, Buckinghamshire, UK) and a LAS-4000 Imager (GE Healthcare). The intensity of SIRT1 expression bands was quantitatively measured using ImageQuant TL software (GE Healthcare) and normalized with that of *β*-actin. When the SIRT1:*β*-actin intensity ratio in the cancerous tissues was more than 2 times, higher than that in the noncancerous tissues by ImageQuant TL quantification, SIRT1 was considered highly expressed.

### IHC

IHC studies were performed as previously described [24]. The primary antibodies used were to SIRT1 (rabbit pAb, HPA006295; ATLAS antibody, Stockholm, Sweden; 1:200), DBC1/p30 (rabbit pAb, IHC-00135; Bethyl Laboratories, Montgomery, TX; 1:500), histone H4K16Ac (rabbit mAb, EPR1004, Gen Tex, San Antonio, TX; 1:100), histone H3K9Ac (rabbit mAb, T.69.2, Thermo Scientific, 1:400), and p53 (mouse mAb, DO7, Dako, Glostrup, Denmark; 1:50). Staining was evaluated on the basis of the positively stained cell percentage and staining intensity. The percentage of positively stained cells was divided into five grades (percentage scores) as follows: 10% (0), 10–25% (1), 25–50% (2), 50–75 (3), and 75% (4). A score of zero was considered to reflect low expression, whereas scores of 1–3 indicated high expression. IHC expression was independently evaluated by 3 pathologists, and a score was assigned only when they all agreed. The expression of DBC1, histone H3K9Ac, histone H4K16Ac, and p53 was also evaluated using the same methodology.

### Statistical analysis

Statistical analyses were performed using the Statistical Package for the Social Sciences (SPSS) version 19.0 (IBM SPSS, Chicago, IL). The associations among the high expression of SIRT1, DBC1, histone H4K16Ac, histone H3K9Ac, and p53 (western blot and IHC) and clinicopathological variables were determined using the *χ*^2^ test (two-tailed). A survival analysis was conducted for all patients. Outcomes were measured in terms of cancer-specific survival. The cause of death was determined by the attending physicians based on patient records and/or death certificates. The Kaplan–Meier method was used to estimate cancer-specific survival, whereas the log-rank test was employed to compare survival between groups. Multivariate analyses were performed using Cox's proportional hazard model.

## Results

### Western blot analysis of paired frozen samples

**Figure 1:**
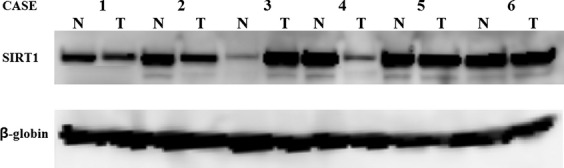
Western blotting of SIRT1 expression in gastric cancer and non-neoplastic gastric mucosa. Clear single bands of SIRT1 around 110 kDa and of *β*-globin around 44 kDa were found.

Fifty-one cancerous and noncancerous pairs of frozen samples were inspected for high SIRT1 expression by western blot analysis (Fig. 1). Of these, 20 (39.2%) cases exhibited high SIRT1 expression. High SIRT1 expression was significantly associated with several poor prognostic factors such as advanced tumor progression (*P* < 0.001), positive lymphatic invasion (*P* = 0.01), positive venous invasion (*P* < 0.001), and advanced stage (*P* = 0.03). Additionally, western blot results for high expression of SIRT1 were closely related to those obtained from IHC studies (*P* = 0.01).

### Clinicopathological variables of cohort gastric cancer patients

Of the 557 patients included, there were 391 men and 166 women with a mean age of 62.0 ± 11.2 years. Among the adenocarcinoma cases, 90 were well-differentiated tumors, 130 were moderately differentiated, and 220 were poorly differentiated. One hundred and seventeen cases could not be categorized owing to the absolute requirement of consensus among the 3 pathologists. Two hundred and twelve cases were negative for lymph node metastasis, whereas 345 were positive. Five hundred and twenty-two cases were negative for distant metastasis and 35 were positive. There were 314 cases of early disease stage, including 273 stage I and 41 stage IIABC cases, whereas the remaining 243 cases were of advanced stage, including 117 stage IIIABC and 126 stage IVAB cases (Table 1).

**Table 1:** Correlation between high expression of SIRT1, DBC1 and H4K16Ac, and clinico-pathological variables.

Clinico-pathological variables	Cases	SIRT1+, *N* (%)	*P*-value	DBC1+, *N* (%)	*P*-value	H4K16Ac+, *N* (%)	*P*-value
Age							
<65	319	191 (60)	0.245	249 (81)	0.565	101 (32)	0.058
≥65	238	154 (65)		189 (83)		92 (40)	
Gender							
Male	391	247 (63)	0.358	306 (81)	0.673	153 (40)	<0.0001*
Female	166	98 (59)		132 (83)		40 (25)	
Histologic							
Differentiation	90	51 (57)	0.123	74 (84)	0.820	52 (59)	<0.0001*
Well	130	91 (70)		109 (84)		61 (48)	
Mod.	220	144 (65)		174 (82)		61 (29)	
Por.							
Tumor status							
T1-2 (M, SM, MP)	318	187 (59)	0.079	262 (86)	0.001*	129 (42)	<0.0001*
T3-4 (SS, SE, SI)	239	158 (66)		176 (75)		42 (27)	
Lymphatic invasion							
Negative	306	177 (58)	0.028*	246 (85)	0.028*	121 (41)	0.005*
Positive	251	168 (67)		192 (77)		72 (29)	
Venous invasion							
Negative	295	169 (57)	0.016*	240 (86)	0.008*	107 (37)	0.354
Positive	262	176 (67)		198 (77)		86 (34)	
Lymph node status							
Negative	212	83 (39)	0.014*	249 (86)	0.002*	118 (75)	0.012*
Positive	345	172 (50)		189 (76)		40 (30)	
Distant metastasis							
Negative	522	321 (64)	0.404	414 (82)	0.043*	182 (36)	0.593
Positive	35	24 (69)		24 (69)		11 (31)	
Stage							
Early (I, II)	314	184 (59)	0.065	257 (86)	0.002*	126 (41)	0.001*
Advanced (III, IV)	243	161 (66)		181 (76)		67 (28)	

* *P*-values <0.05 indicate statistical significance.

### IHC findings of cohort gastric cancer patients

**Figure 2:**
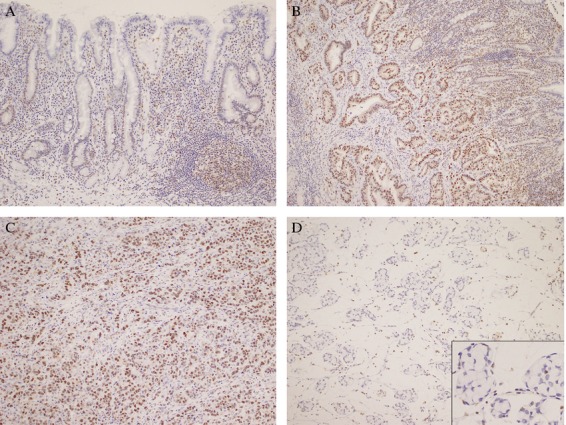
Histology and SIRT1 IHC of gastric cancer and normal gastric mucosa. (A) Note that the majority of the normal gastric foveolar cells are negative for SIRT1 in the nuclei, whereas only some of the cells in the foveolar neck region are positive for SIRT1. Germinal center B-lymphocytes are strongly positive. (B, C) Note that the majority of the tumor cells (well-differentiated adenocarcinoma [B], poorly differentiated adenocarcinoma [C]) are positive for SIRT1 in the nuclei. (D) Majority of mucinous adenocarcinoma and signet ring cells do not show positivity, but some cancer cells show clear nuclear positivity, as seen in inset.

Representative IHC results on SIRT1 expression are shown in Figure 2. Cells with high SIRT1 expression tended to be detected in the lower third of the foveolar duct epithelium, which was overlapping with the proliferative zone, in the normal gastric mucosa. Although most SIRT1 high expression cases exhibited nuclear staining, some had both nuclear and cytoplasmic staining. Additionally, inflammatory cells and germinal center lymphocytes also tended to be positive for SIRT1. DBC1, H4K16Ac, and H3K9Ac but not p53 showed lower grades of IHC positivity in inflammatory cells. However, the overexpression was still readily detected. High expression of SIRT1 was significantly associated with lymphatic invasion (*P* = 0.028), vessel invasion (*P* = 0.016), and lymph node metastasis (*P* = 0.014) and tended to be associated with more advanced disease stages (Table 1). Thus, SIRT1 high expression was generally associated with poor prognostic factors. In contrast, high expression of DBC1 was inversely associated with tumor progression (*P* = 0.001), lymphatic invasion (*P* = 0.028), venous invasion (*P* = 0.008), lymph node metastasis (*P* = 0.002), distant metastasis (*P* = 0.043), and advanced stage (*P* = 0.002) (Table 1). Furthermore, high expression of H4K16Ac was significantly associated with male sex (*P* < 0.001) and inversely associated with the histologic malignant grade (*P* < 0.001), tumor progression (*P* < 0.001), lymphatic invasion (*P* = 0.005), lymph node metastasis (*P* = 0.012), and advanced stage (*P* = 0.001), which was similar to the results of DBC1 (Table 1). Similarly, high expression of H3K9Ac was also significantly associated with male sex (*P* = 0.011) and inversely associated with the histologic malignant grade (*P* < 0.001), tumor progression (*P* = 0.025), and advanced stage (*P* = 0.016) (Table 1). p53 positivity was associated with an older age (*P* = 0.012), the male sex (*P* = 0.004), tumor progression (*P* = 0.023), lymphatic invasion (*P* < 0.0001), vessel invasion (*P* < 0.0001), lymph node metastasis (*P* = 0.008), and advanced stage (*P* = 0.029).

Table 2 demonstrates the correlations among SIRT1, DBC1, H4K16Ac, H3K9Ac, and p53 expressions, which were mutually related.

**Table 2:** Reciprocal correlation among SIRT1, DBC1, H4K16Ac, H3K9Ac, and p53.

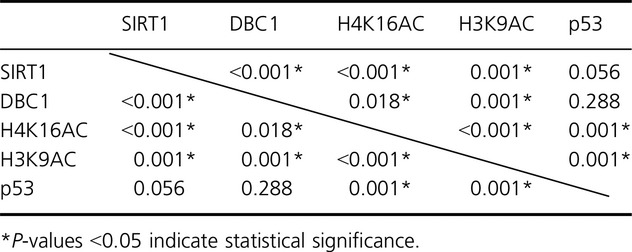

### Cancer-specific survival assessed by the Kaplan–Meier method

**Figure 3:**
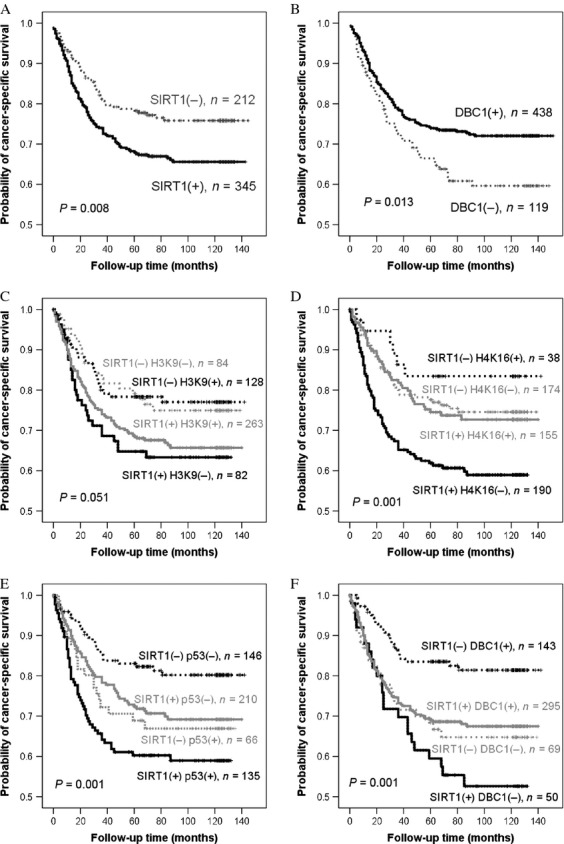
Kaplan–Meier curves of cancer-specific survival time correlated with SIRT1, DBC1, H4K16Ac, H3K9Ac, and p53 expression. (A) Kaplan–Meier survival curves showed high SIRT1 expression to be a worse prognostic indicator with statistical significance. (B) Kaplan–Meier survival curves demonstrated that high DBC1 expression was a significant favorable prognostic indicator. (C) Kaplan–Meier survival curves indicated SIRT1 (−) and DBC1 (+) to be favorable prognostic indicator. (D) Kaplan–Meier survival curves showed that the SIRT1 (−) and H4K16Ac (+) group had very favorable prognosis. (E) There was a close tendency (*P* = 0.051) between SIRT1 (−) and H3K9Ac (+) versus SIRT1 (+) and H3K9Ac (−). (F) Kaplan–Meier survival curves showed that the SIRT1 (+) and p53 (+) group had a worse prognosis.

The observed survival duration ranged from 6 to 142 months (median, 69 months). High expression of SIRT1 was associated with a poor cancer-specific survival prognosis by both univariate (*P* = 0.007) and multivariate (*P* = 0.05) analyses (Table 3). DBC1 and H4K16Ac expressions were linked to a more favorable prognosis by univariate analysis (*P* = 0.014 and *P* = 0.037, respectively) but not multivariate analysis. Additionally, p53 was associated with a poor prognosis only by univariate analysis (*P* = 0.001). Furthermore, our results suggested that SIRT1, DBC1, H4K9Ac, and p53 were excellent prognostic indicators when combined properly (Fig. 3).

**Table 3:** Univariate and multivariate cox regression analyses for cancer-specific survival endpoints in the overall study population in SIRT1, DBC1, H4K16 Ac, H3K9 Ac, and p53.

Factors	Univariate analysis			Multivariate analysis		
	
	RR	(95% CI)	*P*-value	RR	(95% CI)	*P*-value
Age	1.196	0.879–1.627	0.254			
Gender	0.839	0.594–1.186	0.839			
Histologic differentiation	0.460	0.275–0.771	0.003*	0.634	0.374–1.074	0.090
Tumor progression	15.263	9.542–24.413	<0.001*	5.395	3.192–9.121	<0.001*
Lymph node metastasis	11.875	7.498–18.805	<0.001*	3.223	1.884–5.512	<0.001*
Lymphatic	6.234	4.285–9.071	<0.001*	1.215	0.798–1.956	0.363
Venous invasion	8.107	5.358–12.264	<0.001*	1.995	1.257–3.164	0.003*
SIRT1 expression	1.586	1.133–2.220	0.007*	1.396	1.007–1.956	0.050*
DBC1 expression	0.652	0.463–0.916	0.014*	0.962	0.682–1.358	0.828
H4K16Ac expression	0.698	0.497–0.979	0.037*	0.814	0.576–1.151	0.245
H3K9Ac expression	0.997	0.714–1.393	0.985	1.076	0.768–1.507	0.672
p53 expression	1.669	1.228–2.270	0.001*	1.279	0.935–1.750	0.123

CI, Confidence interval; RR, Relative risk.

* *P*-values <0.05 indicate statistical significance.

## Discussion

In this study, we investigated the expression of SIRT1 in gastric cancer patients and its association with clinicopathological factors and outcomes. Our results revealed that SIRT1 expression, as determined by either western blot analysis or IHC, correlated with tumor progression and was an independent predictor of a poor prognosis in gastric cancer patients, which were in agreement with previously reported findings [15, 25]. One of the reasons for such a correlation may involve the oncogenic role of SIRT1 via deacetylation of TSGs including *p53*, resulting in the loss and/or downregulation of TSG and thereby the promotion of prolonged cell survival. However, SIRT1 also functions through various oncogenic processes and has many catalytic partners [9]. There are 3 main ways in which SIRT1 can act as an oncogene. First, in tumor cells, SIRT1 localizes to the promoters of TSGs, including *E-cadherin*, *SFRPs*, *MLH1*, and *p27*, that are aberrantly hypermethylated, leading to their transcriptional repression and subsequent unregulated cell proliferation [6]. Second, the recruitment of SIRT1 to its target gene promoter results in deacetylation of histone proteins at H1K26Ac, H4K16Ac, and H3K9Ac as well as methylation at H3 lysine 9 (H3K9triMe or H3K9diMe) and H3 lysine 79 (H3K79diMe). Such acetylation and methylation by SIRT1 suppress rRNA transcription, resulting in the inhibition of apoptosis [9]. Third, SIRT1 also deacetylates nonhistone proteins, such as transcription factors, DNA repair proteins, and signaling factors [9]. However, it is generally accepted that SIRT1 has a dual role of a TSG and an oncogene [20] as it also participates in DNA damage repair and genomic integrity maintenance [26, 27]. In fact, we previously reported that head and neck squamous cell carcinoma (HNSCC) patients who exhibited SIRT1 expression often experienced tumor regression and had a favorable prognosis [28]. In addition to the complicated dual functions of SIRT1, contradicting results have been reported in IHC studies of gastric cancer [15, 29, 25]. Therefore, in this study, SIRT1 expression was evaluated using 2 different methods (western blot analysis and IHC), which demonstrated consistent results.

DBC1 acts as a native inhibitor of SIRT1 and promotes p53-mediated apoptosis through specific inhibition of SIRT1 [12, 13]. Our results indicated that DBC1 expression assessed by IHC was associated with tumor regression and a favorable prognosis. Additionally, a combination of low DBC1 and high SIRT1 expression was significantly associated with a poor prognosis. Both contradicting [15] and similar [29] results have been reported in gastric cancer studies. In breast cancer, both SIRT1 and DBC1 expressions were associated with tumor progression and a poor prognosis [14], whereas another study indicated that SIRT1 and DBC1 expressions were associated with favorable and unfavorable clinicopathological factors, suggesting their pleiotropic functions as a potential tumor promoter and tumor suppressor during tumorigenesis [25]. In HNSCC, both SIRT1 and DBC1 expressions were associated with tumor regression and a favorable prognosis, despite a dissociation between transcriptional and translational levels [14]. If we simply assume that SIRT1 is a tumor promoter, whereas DBC1 a tumor suppressor counteracting SIRT1, the relative abundance of SIRT1 to that of DBC1 should be elevated in cancers. However, we previously reported that the above simple assumption was not valid in colon cancer [30]. Excessive SIRT1 activity due to low DBC1 expression might be unfavorable for cancer cell growth. Alternatively, DBC1 might contribute to the growth or survival of cancer cells independently of SIRT1, as DBC1 interacts with various proteins including retinoic acid receptor *α*, estrogen receptor *α* and *β*, androgen receptor, SUV39H1 methyltransferase, HDAC 3, and BRCA1, and the interaction between DBC1 and SIRT1 can be lost in some cases [31, 32].

SIRT1 expression was positively correlated with p53 expression but negatively correlated with H4K16Ac and H3K9Ac expression. p53 expression was linked to a poor prognosis presumably via a collaboration with SIRT1. Acetylation of the H4 tail seems to be the most important histone deposition to newly replicated DNA and in chromatin structure, whereas histone H3 modifications might have evolved to ensure the proper control of gene expression. This is suggested by the considerably higher frequency of posttranscriptional modifications found in the *N*-terminal tail of histone H3 relative to that of H4 and the variety of histone variants discovered to date in contrast to a single H4 isoform [19]. Acetylation of histones H4 and H3 exhibits distinct functional and temporal patterns. Most of histone H4 acetylation is cell cycle-dependent and peaks at the replicative S phase, whereas global H3 acetylation levels do not seem to vary [19]. Of the 4 possible lysine residues (K5, 8, 12, and 16) for acetylated in the H4 *N*-terminal tail, K16 is the most frequently acetylated in eukaryotes and its acetylated form is a marker of actively transcribed chromatin. Acetylated K16 is present in ∼60% of all H4 molecules present in mammalian cells [19]. The results of this study may indicate that acetylated H4K16 and H3K9 are linked to the totally stable state or resistance to the oncogenic role of SIRT1.

In conclusion, we found that high expression of SIRT1 was closely associated with progression and prognosis in gastric cancer patients. The findings on DBC1, H4K16Ac, and H3K9Ac also suggested that SIRT1 acted as an oncogene and thus might be a potential target for HDAC inhibitor treatment in gastric cancer patients.
